# Contained sinus of Valsalva hematoma: an unusual entity leading to acute heart failure

**DOI:** 10.1007/s10554-024-03154-3

**Published:** 2024-06-14

**Authors:** Pietro Costantini, Damiano Fedele, Alessandro Carriero, Marco Guglielmo

**Affiliations:** 1 Radiology Department, Azienda Ospedaliera Universitaria Maggiore della Caritá di Novara, University of Eastern Piedmont, Novara, Italy; 2 Department of Medical and Surgical Sciences-DIMEC, Alma Mater Studiorum, University of Bologna, 40138 Bologna, Italy; 3 Division of Heart and Lungs, Department of Cardiology, Utrecht University, Utrecht University Medical Center, Utrecht, The Netherlands

**Keywords:** Sinus of valsalva aneurysm, CT, Computed tomography, Intramural hematoma, Ischemia

A 67-year-old man with a history of treated type A aortic dissection and mechanical aortic valve prosthesis (AVP) underwent computed tomography (CT) for follow-up. CT showed normal AVP and a sinus of Valsalva aneurysm (SVA) (Panels A and B) without significant stenosis of the coronary arteries (Panel C). Three days later the patient presented to the emergency department with shortness of breath and typical chest pain. EKG showed non-specific repolarization abnormalities. Laboratory test revealed elevated high-sensitivity troponins and D-dimer. Chest CT showed a hyperdense crescent sign in the basal scan, consistent with an intramural hematoma (IH) of the SVA (Panels D, E, and F), and ab extrinseco compression of the left main coronary artery (LMCA) by the IH (Panels E and I) leading to diffuse subendocardial ischemia (Panel G, white arrowheads) with signs of acute heart failure (Panel H). The patient refused surgery against medical advice. A follow-up CT performed after 14 days revealed resolution of the IH and of the compression on LMCA (Panels L and M) with no signs of ischemia (Panel N).

IH of the SVA is a very rare complication with scarce literature guiding the management. CT provides a comprehensive assessment of size and potential complications.
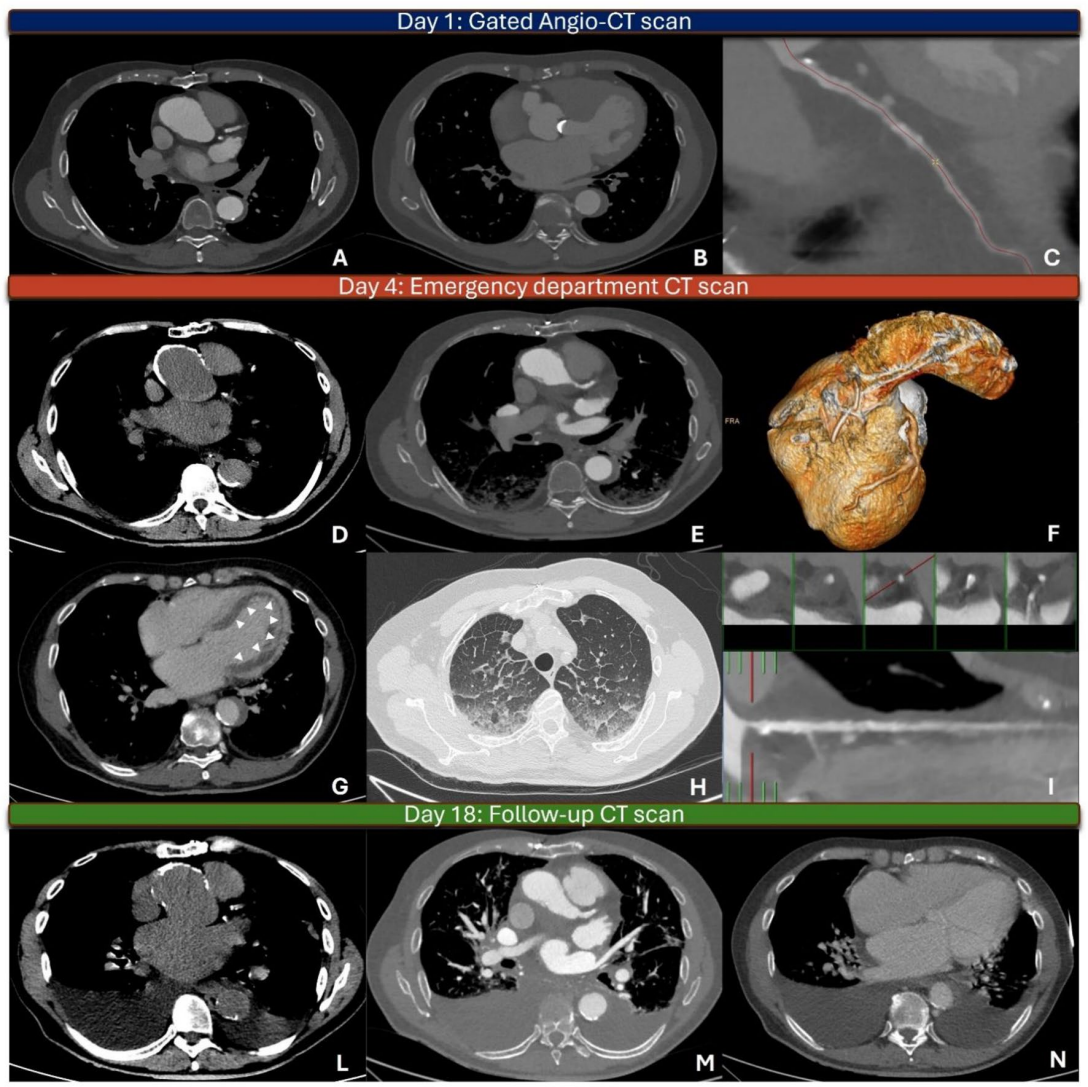


## Data Availability

No datasets were generated or analysed during the current study.

